# Effects of partial silencing of genes coding for enzymes involved in glycolysis and tricarboxylic acid cycle on the enterance of human fibroblasts to the S phase

**DOI:** 10.1186/s12860-015-0062-8

**Published:** 2015-05-28

**Authors:** Aleksandra Konieczna, Aneta Szczepańska, Karolina Sawiuk, Grzegorz Węgrzyn, Robert Łyżeń

**Affiliations:** Department of Molecular Biology, University of Gdańsk, Wita Stwosza 59, 80-308, Gdańsk, Poland

**Keywords:** Glycolysis, Tricarboxylic acid cycle, DNA replication control, S phase of the cell cycle, Human fibroblasts

## Abstract

**Background:**

Previously published reports indicated that some enzymes of the central carbon metabolism (CCM), particularly those involved in glycolysis and the tricarboxylic acid cycle, may contribute to regulation of DNA replication. However, vast majority of such works was performed with the use of cancer cells, in the light of carcinogenesis. On the other hand, recent experiments conducted on bacterial models provided evidence for the direct genetic link between CCM and DNA replication. Therefore, we asked if silencing of genes coding for glycolytic and/or Krebs cycle enzymes may affect the control of DNA replication in normal human fibroblasts.

**Results:**

Particular genes coding for these enzymes were partially silenced with specific siRNAs. Such cells remained viable. We found that silencing of certain genes resulted in either less efficient or delayed enterance to the S phase. This concerned following genes: *HK2, PFKM, TPI, GAPDH, ENO1, LDHA, CS1, ACO2, SUCLG2, SDHA, FH* and *MDH2*. Decreased levels of expression of *HK2, GADPH, CS1, ACO2, FH* and *MDH2* caused also a substantial impairment in DNA synthesis efficiency.

**Conclusions:**

The presented results illustrate the complexity of the influence of genes coding for enzymes of glycolysis and the tricarboxylic acid cycle on the control of DNA replication in human fibroblasts, and indicate which of them are especially important in this process.

**Electronic supplementary material:**

The online version of this article (doi:10.1186/s12860-015-0062-8) contains supplementary material, which is available to authorized users.

## Background

DNA replication is an essential processes in every cellular organism. Its precise regulation is crucial for adequate inheritance of the genetic material by daughter cells, and thus, proper functions of cells and organisms. The general scheme of DNA replication is common in prokaryotic and eukaryotic cells, however, these processes differ significantly in details. Nevertheless, it was indicated that principles of some regulatory mechanisms may be common, or at least similar, in both types of cells (for reviews see [1–3]).

Apart from involvement of specific proteins dedicated solely to control DNA replication, it appeared that enzymes which primary functions were ascribed to other processes can also play important roles in the regulation of genome duplication. Particularly, a new light on this problem was shed by recent studies on bacterial models. It was demonstrated that a direct link exists between central carbon metabolism and DNA replication regulation. Namely, effects of mutations in genes coding for *Bacillus subtilis* primase, helicase or lagging strand DNA polymerase could be specifically suppressed by mutations in genes encoding enzymes catalyzing terminal reactions of glycolysis (*pgk*, *pgm*, *eno*, *pykA*) [4]. In *Escherichia coli*, effects of mutations in genes coding for the α subunit of DNA polymerase III, DNA polymerase III β clamp, and the primase were suppressed by deletions of genes coding for enzymes involved in glycolytic, acetate overflow and pentose-phosphate pathways [5]. Moreover, temperature-sensitive phenotype of a mutation in the *dnaA* gene, coding for the replication initiator protein, was overcome by deletions of *pta* and *ackA* genes, coding for enzymes comprising the acetate overflow mechanism [6].

Although the studies mentioned above were conducted on bacterial models, recent analyse of previously published reports suggested that somewhat similar phenomenon might occur in eukaryotes. Additional roles of enzymes catalyzing reactions of glycolysis and tricaboxylic acid cycle were reported previously, and some of them include regulation of transcription, DNA binding and involvement in carcinogenesis (summarized and discussed in [7, 8]). Therefore, one might speculate that the direct link between central carbon metabolism and DNA replication is not restricted to bacterial cells, but could operate also in eukaryotes, including humans. On the other hand vast majority of such studies on human cells were performed with the use of cancer cell lines. Beside many advantages of the use of such lines, there are also drawbacks when considering regulatory mechanisms of DNA replication, as cancer cells have serious disturbances in the control of this process. Moreover, most studies concentrated on single enzymes, thus, different kinds of experiments were performed for particular genes and proteins. Therefore, the aim of this work was to assess the effects of silencing of genes coding for enzymes involved in all steps of glycolysis and tricarboxylic acid cycle on DNA replication in human non-cancer cells. As a model, we have chosen a human dermal fibroblast cell line, as a representative of cells that actively divide throughout the human life, while being non-transformed.

## Results

### Silencing of genes coding for enzymes involved in glycolysis and tricarboxylic acid cycle

Human dermal fibroblasts, line HDFa, were used in all experiments. To silence the expression of genes encoding enzymes involved in glycolysis and tricarboxylic acid cycle, specific siRNAs were employed. Following genes were subjected to silencing: *HK2* (coding for hexokinase 2), *GPI* (coding for phosphoglucose isomerase)*, PFKM* (coding for phosphofructokinase M)*, ALDOA* (coding for diphosphate aldolase A)*, TPI1* (coding for triosephosphate isomerase)*, GAPDH* (coding for glyceraldehyde 3-phosphate dehydrogenase)*, PGK1* (coding for 3-phosphoglycerate kinase 1)*, PGAM1* (coding for phosphoglycerate mutase 1)*, ENO1* (coding for α-enolase)*, PKM* (coding for pyruvate kinase M)*, LDHA* (coding for lactate dehydrogenase A)*, CS1* (coding for citrate synthase 1)*, ACO2* (coding for aconitase 2)*, IDH2* (coding for isocitrate dehydrogenase 2)*, IDH3B* (coding for isocitrate dehydrogenase 3B)*, OGDH* (coding for α-ketoglutarate dehydrogenase)*, SUCLG2* (coding for GDP-forming succinyl-CoA synthetase 2)*, SDHA* (succinate dehrydrogenase complex, subunit A)*, FH* (coding for fumarase) and *MDH2* (coding for malate dehydrogenase 2).

**Fig. 1:**
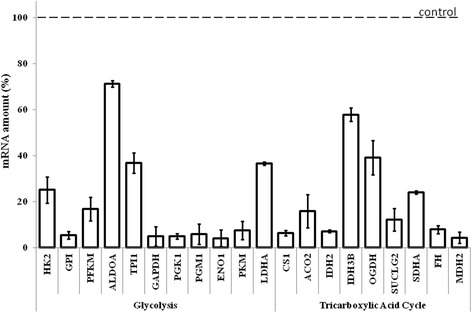
Levels of mRNAs of genes coding for glycolytic and tricarboxylic acid cycle enzymes in human dermal fibroblast cells treated with siRNAs. Cells were seeded in 6-well plates, and transfected with siRNAs. After 72 h incubation, total RNA was purified and the level of mRNA was estimated by qPCR analysis. Presented results are mean values from at least three independent experiments, with error bars indicating SD. In each experiment, mRNA level measured in untreated cells was used as a control value (100 %, dashed line). In all experiments, statistically significant differences (p < 0.05 in the t-test) were found relative to the control

**Fig. 2:**
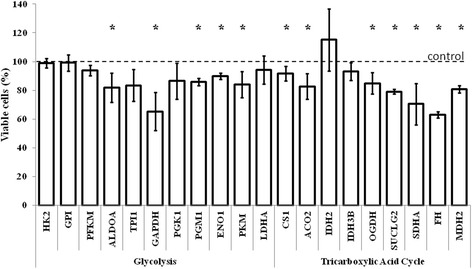
Viability of human dermal fibroblasts after silencing of genes coding for glycolytic and tricarboxylic acid cycle enzymes. Cells were seeded in 6-well plates, transfected with siRNAs and synchronized. Following washing, the cells were collected and analyzed by flow cytometry. Presented results are mean values from at least three independent experiments, with error bars indicating SD. In each experiment, mRNA level measured in untreated cells was used as a control value (100 %, dashed line). Statistically significant differences relative to the control are indicated by asterisks

Particular siRNAs caused various inhibition of expression of specific genes. The levels of different transcripts were from about 70 % to less than 10 %, relative to the control (non-treated) cells (Fig. 1). Nevertheless, the cells remained viable, with little or moderate effects of the treatment on the number of alive cells in the culture. The most pronounced effects were observed for silencing of *GAPDH* and *FH* genes, where 60 % of cells survived siRNA-mediated expression impairment (Fig. 2).

### Enterance to the S phase following gene silencing

**Fig. 3:**
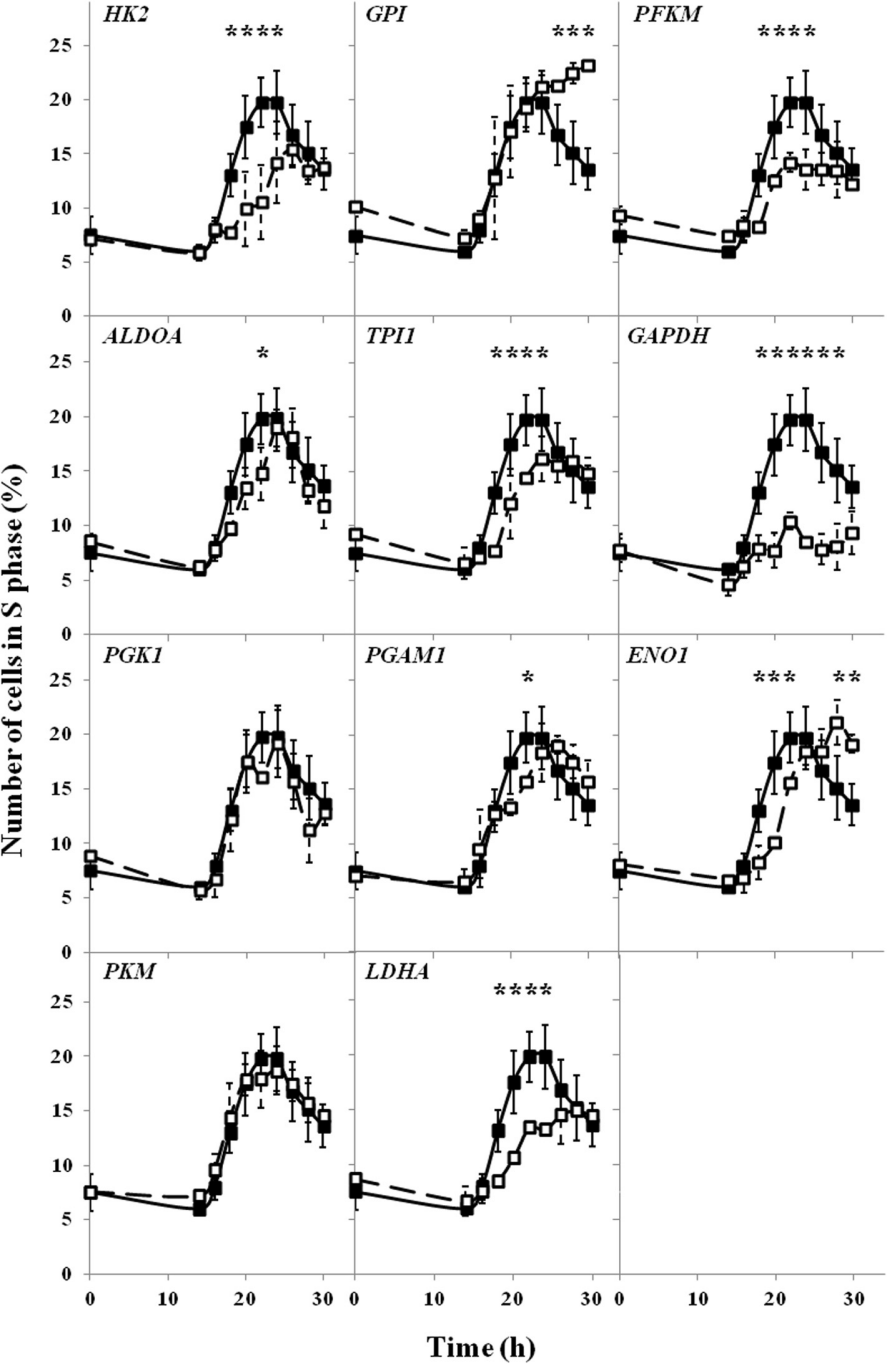
Effects of siRNA-mediated silencing of glycolityc genes on enterance of cells in S phase. Cells were seeded on Petri dishes, transfected with siRNA specific for indicated gene (□) and synchronized. Analogous experiments without siRNA were treated as controls (■). After cell cycle releasing, the cells were collected every two hours, starting from 14 h, and analyzed by flow cytometry. Presented results are mean values from at least three independent experiments, with error bars indicating SD. Statistically significant differences relative to the control are indicated by asterisks

The time and efficiency of the enterance of the cells to the S phase following silencing of expression of particular genes were estimated. Two types of effects were observed in cells treated with siRNAs impairing expression of some genes, less efficient or delayed enterance to S phase. When genes coding for enzymes catalyzing reactions of glycolysis were silenced, the less efficient enterance in the S phase, as measured by the percentage of cells in this phase, was observed for fibroblasts with impaired expression of *HK2, PFKM, TPI, GAPDH* and *LDHA*, with the most pronounced effect in the case of *GAPDH* (Fig. 3). Delayed enterance in the S phase, with a similar fraction of cells entering this phase, was observed in fibroblasts with silenced the *ENO1* gene (Fig. 3). Analysis of other phases of the cell cycle under these conditions is presented as additional data [Additional file 1 and 2].

**Fig. 4:**
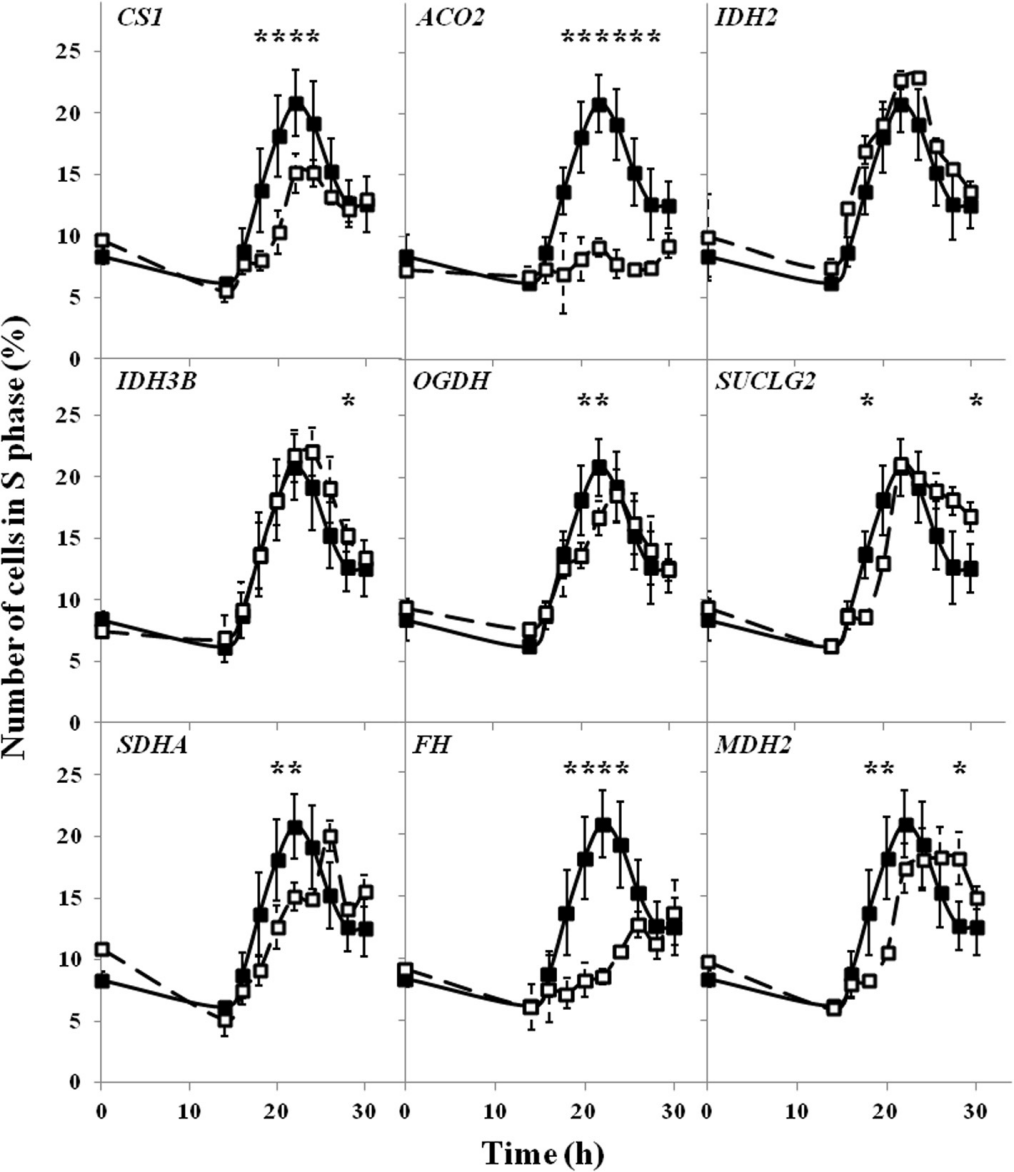
Effects of siRNA-mediated silencing of tricarboxylic acid cycle genes on enterance of cells in S phase. Cells were seeded on Petri dishes, transfected with siRNA specific for indicated gene (□) and synchronized. Analogous experiments without siRNA were treated as controls (■). After cell cycle releasing, the cells were collected every two hours, starting from 14 h, and analyzed by flow cytometry. Presented results are mean values from at least three independent experiments, with error bars indicating SD. Statistically significant differences relative to the control are indicated by asterisks

When the tricarboxylic acid cycle genes were silenced, less efficient enterance to the S phase was observed in cells with impaired expression of *CS1, ACO2, SDHA* and *FH,* with the most pronounced effects in the case of *ACO2* and *FH,* and the delayed enterance occurred in fibroblasts with silenced *SUCLG2* and *MDH2* genes (Fig. 4). Analysis of other phases of the cell cycle under these conditions is presented as additional data [Additional file 3 and 4].

### DNA synthesis in cells with silenced genes

**Fig. 5:**
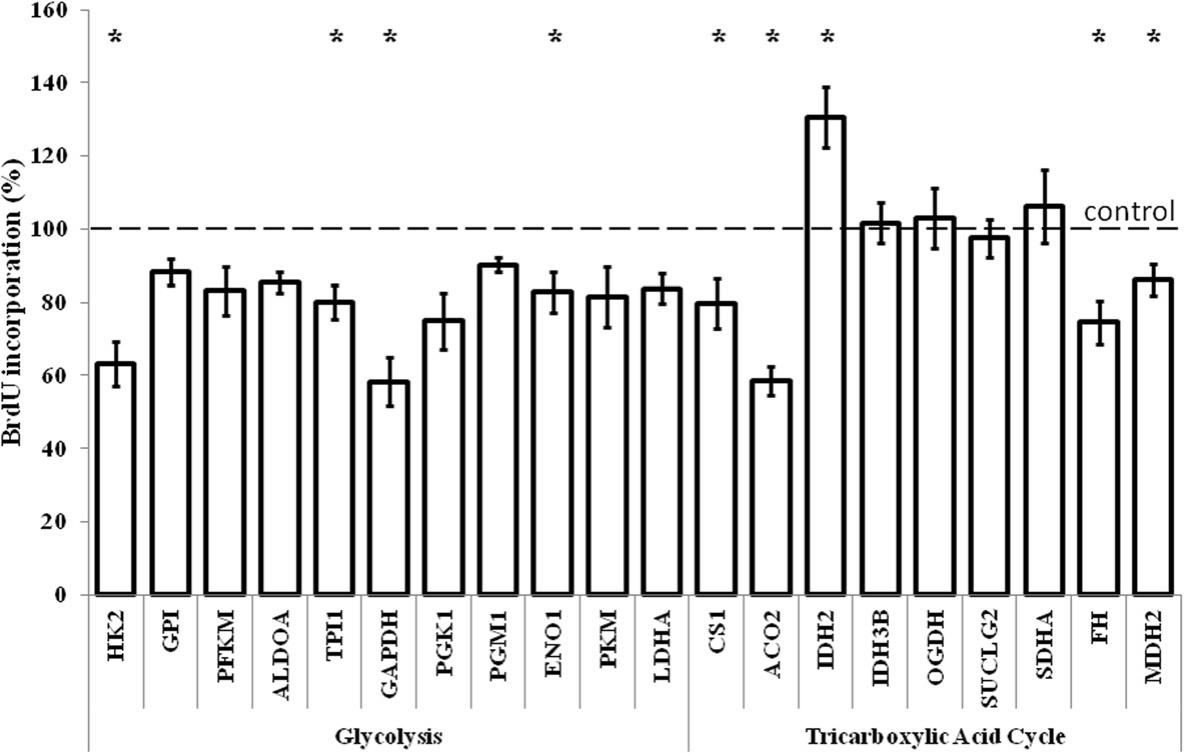
DNA synthesis in human dermal fibroblasts treated with siRNAs. Following siRNA trasfection and synchronization, cells were labeled with BrdU for 24 h. Then, the cells were fixed, and incubated with anti-BrdU antibodies. BrdU incorporation was quantified by a colorimetric reaction (absorbance at 460 nm). Presented results are mean values from at least three independent experiments, with error bars indicating SD. In each experiment, DNA synthesis level measured in untreated cells was used as a control value (100 %, dashed line). Statistically significant differences relative to the control are indicated by asterisks

The results described in the preceding subsection indicated that silencing of several genes coding for enzymes involved in glycolysis and tricarboxylic acids had significant effects on the enterance of human fibroblasts in the S phase. Less effective or delayed enterance of cells in the S phase should imply impairment in DNA replication. To test if DNA synthesis is affected in fibroblasts with assessed genes, we have measured rates of incorporation of bromodeoxyuridine (BrdU) in synchronized cell cultures. In most cases, impairment of DNA synthesis was negligible if any. However, silencing of *HK2, GADPH, CS1, ACO2, FH* and *MDH2* resulted in significantly less efficient incorporation of BrdU into DNA of cultured cells (Fig. 5). Since effects on enterance to S phase were observed in cells with impairment expression of the same genes, the results of measurement of DNA synthesis efficiency corroborate the conclusion made on the basis of those experiments.

## Discussion

**Fig. 6:**
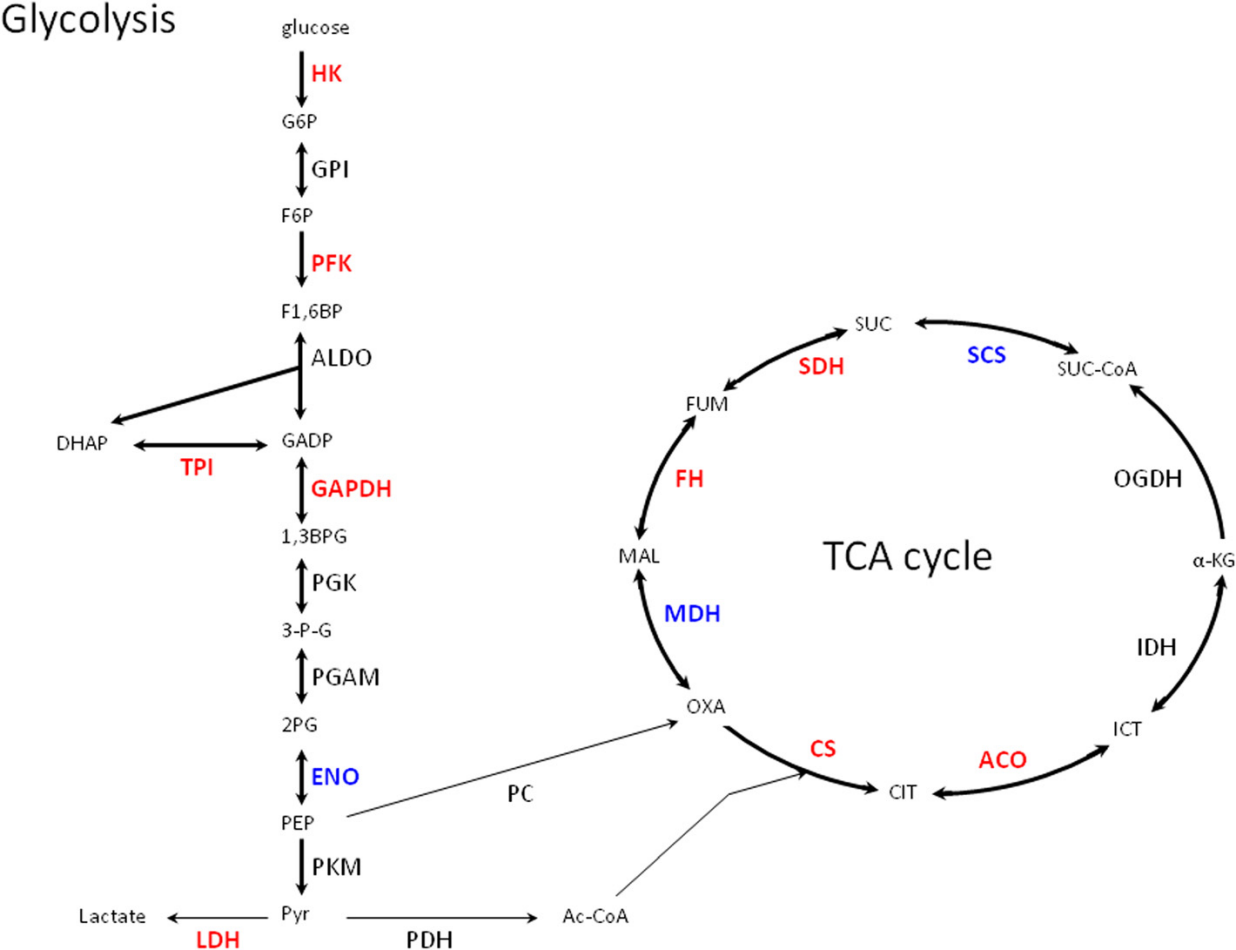
The scheme of glycolysis and the tricarboxylic acid cycle, with indicated enzymes which impaired production, due to silencing of corresponding genes, resulted in less efficient (marked in red **bold** font) or delayed (marked in blue **bold** font) enterance of human dermal fibroblasts to the S phase. Abbreviations: ACO - aconitase; ALDO - fructose-bisphosphate aldolase; CS - citrate synthase; ENO - enolase; FH - fumarase; GAPDH - glyceraldehyde phosphate dehydrogenase; GPI - phosphoglucose isomerase; HK - hexokinase; IDH - isocitrate dehydrogenase; LDH - lactate dehydrogenase; MDH - malate dehydrogenase; OGDH - α-ketoglutarate dehydrogenase; PC - pyruvate carboxylase; PDH - puryvate dehydrogenase; PFK - phosphofructokinase; PGK - phosphoglycerate kinase; PGAM - phosphoglycerate mutase; PKM - pyruvate kinase; SCS - succinyl-CoA synthetase; SDH - succinate dehydrogenase; TPI - triosephosphate isomerase. Abbreviations of metabolite names are as follows: 1,3BPG - 1,3-bisphosphoglycerate; 2PG - 2-phosphoglycerate; 3-P-G - 3-phosphoglycerate; AC-CoA - Acetyl-CoA; CIT - citrate; DHAP - dihydroxyacetone phosphate; F1,6BP - fructose 1,6-bisphosphate; F6P - fructose 6-phosphate; FUM - fumarate; G6P - glucose 6-phosphate; GADP - glyceraldehyde 3-phosphate; ICT - isocitrate; MAL - malate; OXA - oxaloacetate; PEP - phosphoenolpyruvate; Pyr - pyruvate; SUC - succinate; SUC-CoA - α-ketoglutarate

A direct link between central carbon metabolism and DNA replication has been demonstrated recently in prokaryotic cells (summarized in [1, 9]). Analysis of previously published data led to the hypothesis that enzymes of central carbon metabolism may also be involved in the regulation of DNA replication [7, 8]. However, particular previous reports were usually focused on single enzymes. Moreover, vast majority of works on human cells were performed with cancer-derived cell lines, which may have serious drawbacks when studying the DNA replication control. Therefore, we have performed a complex study, in which expression of genes coding for enzymes involved in all steps of glycolysis and tricarboxylic acid cycle were silenced with the use of specific siRNAs. Interestingly, we found that silencing of certain genes resulted in either less efficient or delayed enterance to the S phase. This concerned following genes: *HK2, PFKM, TPI, GAPDH, ENO1, LDHA, CS1, ACO2, SUCLG2, SDHA, FH* and *MDH2*. Decreased levels of expression of *HK2, GADPH, CS1, ACO2, FH* and *MDH2* caused also a substantial impairment in DNA synthesis efficiency. These effects, with indicated genes’ products in the metabolic pathways, are summarized schematically in Fig. 6.

These results may suggest that central carbon metabolism has a significant direct influence on the regulation of DNA replication through particular enzymes. At this stage of our knowledge it is impossible to deduce a specific mechanism by which these enzymes may link the metabolism to DNA synthesis. However, there are some insights from previous works, summarized below, which might shed some light on this phenomenon.

Impaired expression of *HK2*, coding for hexokinase 2, in cancer-associated fibroblasts (CAF) resulted in a G1 phase cell cycle arrest [10]. Moreover, decreasing of *HK2* expression in laryngeal squamous cell carcinoma (LSCC) reduced proliferation and cell viability by increasing G0-G1 ratio and apoptosis [11]. Our results, indicating a decreased efficiency of the enterance to S phase after silencing of *HK2*, are compatible with those observations. One of isoenzymes of phosphofructokinase, was reported as a metabolic effector involved in the connection between glycolysis, cell proliferation and transformation [12]. In fact, we also observed an impairment in DNA synthesis when *PFKM* expression was down-regulated. Moreover, depletion of GAPDH with RNA interference in human lung carcinoma A549 and UO31 cells stopped cell proliferation, and induced cell cycle arrest in G1 phase [13], which is in accordance to severe inhibition of the enterance into S phase reported here. Enolase could bind to specific DNA sequences and was found in nuclei of various cell types [14],[15]. Silencing of the *ENO1* gene by siRNA inhibited the proliferation of the HCC cell line, which was accompanied by a shortened S phase and elongated G2/M phase of the cell cycle [16]. Downregulation of ENO1 by siRNA inhibited cell migration and invasion in glioma cells. Reduction of ENO1 activity significantly decreased the phosphorylation of PI3K and Akt and reduced level of E-Cadherin, Cyclin D1, and p-Rb [17]. Thus, it is intriguing that we have observed a delay in the enterance to S phase in cells with the partially silenced *ENO1* gene. Lactate dehydrogenase was found in nuclei of mammalian cells, and its possible function in DNA replication was suggested [18],[19]. Knocking down the expression of *LDHA* in human hepatocellular carcinoma cells and pancreatic cancer cells inhibited cell growth dramatically by activation of the apoptosis pathway [20], [21],[22]. In human fibroblasts with the silenced *LDHA* gene, we have observed a decreased number of cells entering the S phase. In cancer cell lines, HeLa and SiHa, a decrease in expression of the *CS1* gene was proportional to the malignancy, but this effect appeared to be linked to disturbed p53 function [23]. On the other hand *CS* knockdown in human ovarian adenocarcinoma cell line SKOV3 and A2780 cells resulted in dysregulation of cell metabolism and downregulation of proliferation by decreasing phosphorylation of the extracellular signal-regulated kinase (ERK), a key component in the control of cell growth, and increasing of CASP7 encoded Caspase 7 involved in the caspase activation cascade responsible for the execution of apoptosis [11]. In accordance to those reports, some negative effects on DNA replication in human fibroblasts with impaired expression of *CS1* were found in this work. Silencing of expression of the *SDHA* gene resulted in a decrease of growth rate of cancer cells [24]. In our experiments, such silencing caused less efficient enterance into S phase. Finally, fumarase has been proposed to act as a tumor suppressor [25]. Knockdown of *FH* and *SDHA/B* genes in HeLA cells led to accumulation of fumarate and succinate, which act as competitive inhibitors of multiple α-ketoglutarate-dependent dioxygenases, including histone and DNA demethylases [26]. Inactivation of histone demethylases, promotes G1 cell-cycle arrest, and induces genes for differentiation by selectively modulating the methylation states of histone H3 at lysines 4 (H3K4) and 9 (H3K9) [27]. In human fibroblasts, we have observed a severe inhibition of the cell cycle at the stage of the S phase enterance under conditions of partial *FH* silencing.

The advantage of this study, in relation to previous works, discussed above in comparison to our work, is that the results presented here were obtained in the same cell line of human dermal fibroblasts. Generally, we have observed two types of the effects of silencing of particular genes: less efficient or delayed enterance to the S phase. Furthermore, reduction in efficiency in DNA synthesis was demonstrated in cells deficient in expression of the same genes as in the case of the less efficient enterance to the S phase. Therefore, the studies reported here provide a complex picture of the effects of a decreased levels of expression of the central carbon metabolism genes on DNA replication in human fibroblasts.

In the light of the general mechanisms of glycolysis, our results indicating different effects caused by silencing of *GPI* or *GAPDH* gene, an increase or severe decrease in efficiency of enterance into S phase, respectively, may be considered as intriguing. Glycolysis is a multi-step process, and each of the steps is performed either by one or multiple enzymes (isoforms). In contrast to the other steps, the second and sixth steps are catalyzed by the only one enzyme, GPI and GAPDH, respectively. GPI is the only enzyme catalyzing the second step of glycolysis, performing the conversion of glucose-6-phosphate to fructose-6-phosphate. Similarly, GAPDH is another bottleneck, the only enzyme catalyzing the sixth glycolytic step (except GAPDHS, a testis-specific isoform of GAPDH). Thus, metabolic effects (glycolysis down-regulation) of silencing *GPI* and *GAPDH* genes might be expected to be similar. Contrary to such presumption, the observed effects on cell proliferation have been completely different when either *GPI* or *GAPDH* were silenced with siRNA, as indicated above. The dramatic decrease in the fraction of cells entering to the S phase, as well as in viable cells count, when expression of *GAPDH* is impaired, indicates that GAPDH plays not only metabolic roles but also may be directly involved in the regulation of cell proliferation. In fact, despite the lack of direct proofs of GAPDH involvement in DNA replication and cell proliferation, this enzyme is considered as a potential cancer therapeutic target [28].

Although we are not able to propose specific mechanism(s) by which enzymes of glycolysis and tricarboxylic acid cycle can influence the regulation of DNA replication, it appears that the link between these metabolic pathways and the control of cell cycle, particularly DNA synthesis, is important. Plausibly, this link is direct through certain enzymes. Alternatively, metabolites which accumulate due to impairment of enzymatic activities might acts as signals in the regulatory processes as it was shown for fumarate and succinate [26]. Irrespective of the detailed molecular mechanisms, it seems that DNA replication in human cells can be specifically regulated in response to the metabolic status of the cell, and there are several steps in glycolysis and tricarboxylic acid cycle which efficiency could be sensed by the DNA replication machinery. Finally, it is interesting that the direct links between central carbon metabolism and DNA replication appear to exist in both eukaryotic and prokaryotic systems (for discussions see [1, 7, 9]). Therefore, one might speculate that sensing the metabolic status of the cell by the cellular replication factory is an evolutionarily old phenomenon, which can be of particular importance for cell physiology.

## Conclusions

Partial silencing of genes coding for enzymes catalyzing particular reactions of glycolysis and the tricarboxylic acid cycle illustrated the complexity of the influence of central carbon metabolism on the control of DNA replication in human fibroblasts. Following genes appear to be especially important in this process: *HK2, PFKM, TPI, GAPDH, ENO1, LDHA, CS1, ACO2, SUCLG2, SDHA, FH* and *MDH2*. These results, together with previously published reports describing the link between central carbon metabolism and DNA replication in bacteria, might suggest that sensing the metabolic status of the cell by the cellular replication factory is an evolutionarily old phenomenon, which can be of particular importance for cell physiology.

## Methods

### Cell cultures

The Human Dermal Fibroblasts, adult HDFa (Cascade Biologics) were cultured in a 5 % CO_2_ humidified atmosphere at 37 °C in Dulbecco’s Modified Eagle’s Medium (DMEM; GIBCO) supplemented with 10 % fetal bovine serum (FBS; GIBCO).

### Cell cycle synchronization

HDFa were subjected to gradual serum deprivation: 5 % FBS for 6 h, 1 % FBS for 6 h and DMEM without FBS for 12 h. The DNA content was analyzed by MuseTM Cell Analyzer. DNA histogram revealed that over 90 % of HDFa were inhibited at G0/G1 phase after starvation. Then, the cells were released into cell cycle by addition of 10 % serum.

### RNA interference

Silencer® Select siRNAs (Small interfering RNAs) were purchased from Life Technology/Ambion. Transfections were performed with HiPerFect Transfection Reagent (Qiagen) as specified by the manufacturer. All targeted enzymes showed maximal knockdown 72 h after transfection.

### mRNA quantitation

1 × 10^5^ cells were seeded in 6-well plates. 72 h after transfection total RNA was extracted with High Pure RNA Isolation Kit (Roche Diagnostics). The cDNA for PCR template was generated by using Transcriptor First Strand cDNA Synthesis Kit (Roche Diagnostics) according to the manufacturer's protocol. Real-time PCR was performed on a LightCyler system 0.2 (Roche Applied Science) by using LightCycler® TaqMan Master Kit. The sequences of primers and housekeeping genes for normalization are presented in Table 1. The choice of particular reference genes was depended on the residual expression level of the tested gene.

**Table 1:** Primers used for real-time qPCR to estimate mRNA levels of particular genes

Gene name:	Primer sequence	Reference genes
*HK2*	F: 5’- CGAGGTCTGAGCAAGGAGAC	GUSB, HPRT
	R: 5’- GTCCGGGGTAGCACACAC	
*GPI*	F: 5’- GCTTTGCTGCGTACTTCCA	TBP, ACTB
	R: 5’- GTCCACACGGGTTCCAGA	
*PFKM*	F: 5’- GCCATCAGCCTTTGACAGA	GUSB, HPRT
	R: 5’- CTCCAAAAGTGCCATCACTG	
*ALDOA*	F: 5’- TCCTCTAGCCCGTGGAATC R: 5’- AAGACGATGGCAGGGATG	TBP, ACTB
*TPI1*	F: 5’- GCTCAGAGCACCCGTATCAT	GUSB, HPRT
	R: 5’- CACAAGGAAGCCATCCACAT	
*GAPDH*	F: 5’- ACGGGAAGCTTGTCATCAAT	TBP, ACTB
	R: 5’- CATCGCCCCACTTGATTTT	
*PGK1*	F: 5’- CTCATGGATGAGGTGGTGAA	TBP, ACTB
	R: 5’- CACAGCAAGTGGCAGTGTCT	
*PGAM1*	F: 5’- AGGCGCTCCTATGATGTCC	TBP, ACTB
	R: 5’- CGATCCTTACTGATGTTGCTGT	
*ENO1*	F: 5’- CAACCAGCTCCTCAGAATTGA	GUSB, HPRT
	R: 5’- GCCAAGGGGTTTCTGAAGTT	
*PKM*	F: 5’- ACCCTCCACTCAGCTGTCC	TBP, ACTB
	R: 5’- CCTGGAGGTGCTGCAGTAGT	
*LDHA*	F: 5’- GGTGGATGTTTACCGTGTGTT	TBP, ACTB
	R: 5’- TGGATCCCAGGATGTGACTC	
*CS*	F: 5’- TCCGACCCTTACCTGTCCTT	TBP, ACTB
	R: 5’- ACTTCCTGATTTGCCAGTCC	
*ACO2*	F: 5’- AGATTGTGTATGGACACCTGGA	HPRT, GUSB
	R: 5’- TACGACTTGCCTCGCTCAAT	
*IDH2*	F: 5’- CCATCATCTGCAAAAACATCC	HPRT, GUSB
	R: 5’- CCAATGGTGATGGGCTTG	
*IDH3B*	F: 5’- GCCCAATCTCTATGGGAACA	TBP, ACTB
	R: 5’- CAGGGACCACACCAGCTC	
OGDH	F: 5’- AGAGTCCCCTTCCCCTGAG	TBP, ACTB
	R: 5’- GCTTCTACCAGGGACTGTCC	
*SUCLG2*	F: 5’- AGCCAGCCAACTTCTTGGA	TBP, ACTB
	R: 5’- GGATGGCTTCAACCTTAGGA	
*SDHA*	F: 5’- CAGCACAGGGAGGAATCAAT	HPRT, GUSB
	R: 5’- CTGCTCCGTCATGTAGTGGA	
*FH*	F: 5’- TGAATGTTTTCAAGCCAATGAT	HPRT, GUSB
	R: 5’- CCACCACGCAGTTTTCTGTA	
*MDH2*	F: 5’- CAGGACCAGCTGACAGCAC	TBP, ACTB
	R: 5’- AGCCTGCTCCGGCTTTAG	

### Cell cycle analysis

Cell cycle analysis was performed utilizing Muse™ Cell Analyzer (Merck Millipore) and following manufacturer’s instruction. Briefly, after the siRNAs transfection and subsequent cell cycle synchronization, the cells were washed with PBS and fixed in 70 % ice-cold ethanol. Cells were collected every two hours starting from 14 h after cell cycle releasing by addition of 10 % serum. After staining with Muse™ Cell Cycle Reagent, the cells were processed for cell cycle analysis.

### Cell counting and viability

Cell counting and viability was determined by using the Muse® Count & Viability Assay Kit (Merck Millipore), according to the manufacturer’s instruction. Briefly, after the transfection with siRNAs, and subsequent cell cycle synchronization, the cells were collected and incubated with Muse™ Count &Viability Reagent. The number of viable cells were counted by using Muse™ Cell Analyzer (Merck Millipore).

### Proliferation assay

Cell proliferation was determined by using the Cell Proliferation ELISA, BrdU (colorimetric) (Roche Diagnostics) Kit according to the manufacturer's instructions. Briefly, cells were seeded onto 96-well plate in an amount of 1000 cells/well. After siRNAs transfection and subsequent synchronization, cells were labeled with BrdU for 24 h. Then, cells were fixed, incubated with anti-BrdU antibodies and the BrdU incorporation was quantified by colorimetric reaction. Absorbance at 450 nm was measured by using an automated microplate reader (Wallac 1420 Multilabel Counter, Perkin Elmer).

## Additional files

Additional file 1: Figure 1. Effects of siRNA-mediated silencing of glycolityc genes on the fraction of cells in G0/G1 phase. Cells were seeded on Petri dishes, transfected with siRNA specific for indicated gene (□) and synchronized. Analogous experiments without siRNA were treated as controls (■). After cell cycle releasing, the cells were collected every two hours, starting from 14 h, and analyzed by flow cytometry. Presented results are mean values from at least three independent experiments, with error bars indicating SD. Statistically significant differences relative to the control are indicated by asterisks.

Additional file 2: Figure 2. Effects of siRNA-mediated silencing of glycolityc genes on the fraction of cells in G2/M phase. Cells were seeded on Petri dishes, transfected with siRNA specific for indicated gene (□) and synchronized. Analogous experiments without siRNA were treated as controls (■). After cell cycle releasing, the cells were collected every two hours, starting from 14 h, and analyzed by flow cytometry. Presented results are mean values from at least three independent experiments, with error bars indicating SD. Statistically significant differences relative to the control are indicated by asterisks.

Additional file 3: Figure 3. Effects of siRNA-mediated silencing of tricarboxylic acid cycle genes on the fraction of cells in G0/G1 phase. Cells were seeded on Petri dishes, transfected with siRNA specific for indicated gene (□) and synchronized. Analogous experiments without siRNA were treated as controls (■). After cell cycle releasing, the cells were collected every two hours, starting from 14 h, and analyzed by flow cytometry. Presented results are mean values from at least three independent experiments, with error bars indicating SD. Statistically significant differences relative to the control are indicated by asterisks.

Additional file 4: Figure 4. Effects of siRNA-mediated silencing of tricarboxylic acid cycle genes on the fraction of cells in G2/M phase. Cells were seeded on Petri dishes, transfected with siRNA specific for indicated gene (□) and synchronized. Analogous experiments without siRNA were treated as controls (■). After cell cycle releasing, the cells were collected every two hours, starting from 14 h, and analyzed by flow cytometry. Presented results are mean values from at least three independent experiments, with error bars indicating SD. Statistically significant differences relative to the control are indicated by asterisks.
