# Supplementary material for: Effects of partial silencing of genes coding for enzymes involved in glycolysis and tricarboxylic acid cycle on the enterance of human fibroblasts to the S phase
Source: BMC Cell Biol. 2015 May 28;16:16. doi: 10.1186/s12860-015-0062-8 (PMC4446904; doi:10.1186/s12860-015-0062-8)
Supplement: Supplementary file 4 — Effects of siRNA-mediated silencing of tricarboxylic acid cycle genes on the fraction of cells in G2/M phase. Cells were seeded on Petri dishes, transfected with siRNA specific for indicated gene (□) and synchronized. Analogous experiments without siRNA were treated as controls (■). After cell cycle releasing, the cells were collected every two hours, starting from 14 h, and analyzed by flow cytometry. Presented results are mean values from at least three independent experiments, with error bars indicating SD. Statistically significant differences relative to the control are indicated by asterisks. [file 12860_2015_62_MOESM4_ESM.pdf]

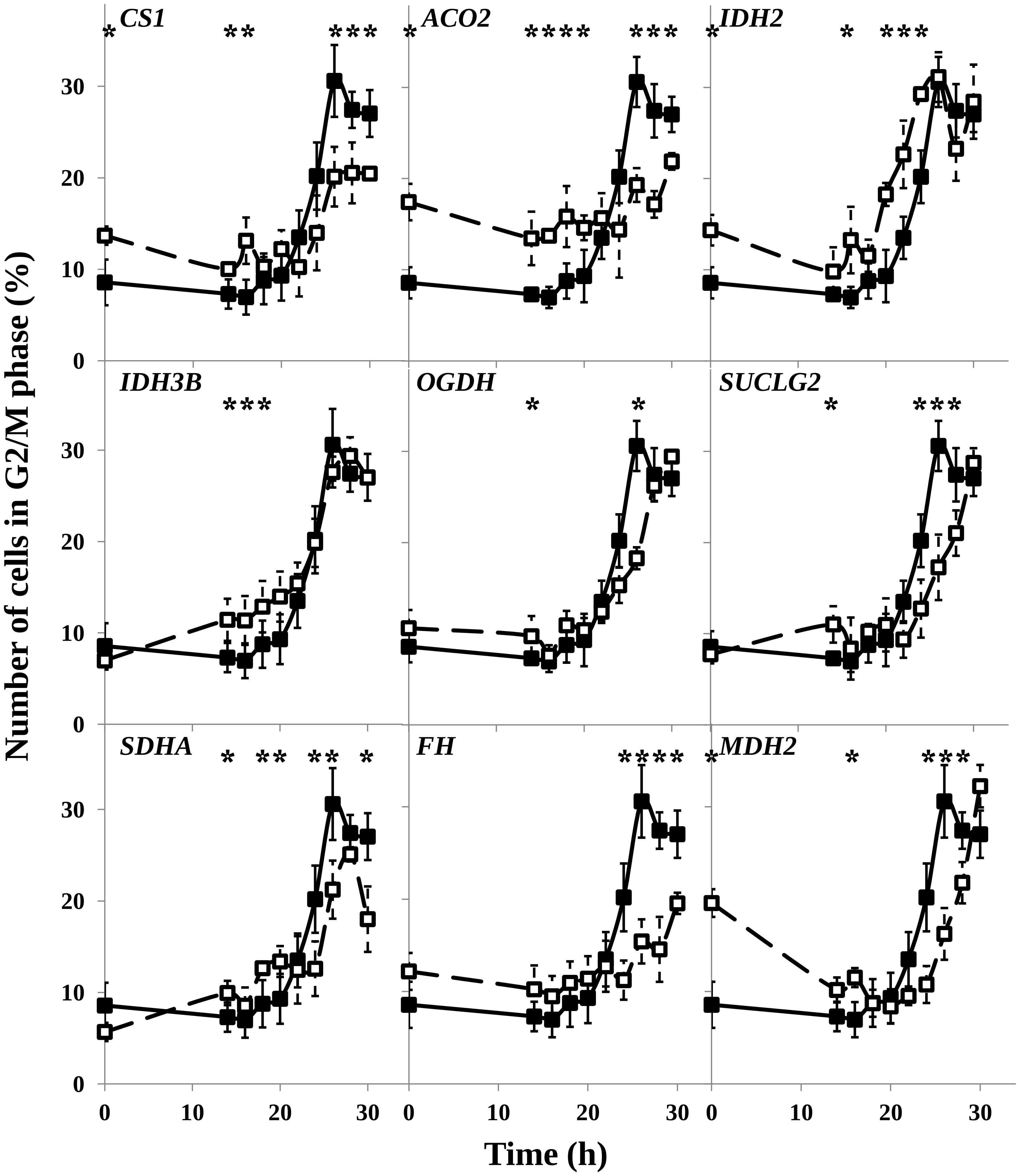

Additional figure 4. Effects of siRNA-mediated silencing of tricarboxylic acid cycle genes on the fraction of cells in G2/M phase. Cells were seeded on Petri dishes, transfected with siRNA specific for indicated gene (□) and synchronized. Analogous experiments without siRNA were treated as controls (■). After cell cycle releasing, the cells were collected every two hours, starting from 14 h, and analyzed by flow cytometry. Presented results are mean values from at least three independent experiments, with error bars indicating SD. Statistically significant differences relative to the control are indicated by asterisks.
